# Adult‐Onset Effmann Type IIA2 Y‐Type Urethral Duplication Presenting as Unexplained Perineal Wetness: A Case Report Highlighting Diagnostic Pitfalls and Tailored Surgical Management

**DOI:** 10.1002/iju5.70174

**Published:** 2026-04-05

**Authors:** Norichika Ueda, Tadahiko Kimura, Kentaro Takezawa, Taigo Kato, Koji Hatano, Yoichi Kakuta, Norio Nonomura, Atsunari Kawashima

**Affiliations:** ^1^ Department of Urology The University of Osaka Graduate School of Medicine Osaka Japan

**Keywords:** adult‐onset congenital anomaly, Effmann type IIA2 Y‐type, perineal urinary leakage, urethral duplication

## Abstract

**Introduction:**

Persistent perineal wetness in adults with normal standard imaging is uncommon and may indicate rare congenital anomalies such as urethral duplication.

**Case Presentation:**

A man in his early 20s presented with a 3‐year history of intermittent perineal wetness of unknown origin. Extensive gastrointestinal and urological investigations were unrevealing. Indigo carmine administration confirmed urinary leakage, prompting focused evaluation. Endoscopic assessment with meticulous perineal inspection under general anesthesia identified a tiny, non‐inflamed perineal opening. Fistulography and cystoscopy demonstrated an accessory tract arising from the prostatic urethra and opening to the perineum, consistent with Effmann type IIA2 Y‐type urethral duplication, extremely rare in adult‐onset. Complete excision was achieved using a combined perineal and laparoscopic transabdominal approach with light‐emitting catheter guidance, preserving urinary continence and erectile function. Symptoms resolved immediately without recurrence.

**Conclusion:**

This case highlighted key diagnostic pitfalls and demonstrates effective surgical management of adult‐onset type IIA2 Y‐type urethral duplication.

## Keynote Message

Persistent perineal wetness in adults with normal imaging should prompt confirmation of urinary leakage and consideration of rare congenital anomalies. Adult‐onset Effmann type IIA2 Y‐type urethral duplication requires an individualized surgical approach to achieve complete excision while preserving function.

## 1 Introduction

Persistent perineal wetness in adults is uncommon and frequently leads clinicians to suspect acquired anorectal or urogenital fistulas [1, 2, 3]. When standard cross‐sectional imaging and endoscopic investigations are normal, diagnosis can be particularly challenging.

Urethral duplication is an exceedingly rare congenital anomaly, usually diagnosed in childhood [4]. Among its subtypes, Effmann type IIA2 (Y‐type) duplication—characterized by an accessory urethral tract branching from the posterior or prostatic urethra and opening ectopically to the perineum—is especially rare in adults [5]. In such cases, the diagnosis may be delayed or even remain unrecognized because symptoms are subtle and routine investigations may fail to identify a small, non‐dilated accessory tract.

Complete excision of the accessory urethra is generally recommended to prevent recurrence of urinary leakage and avoid long‐term complications, including chronic irritation and the potential risk of malignant transformation in residual urethral epithelium [6]. However, in adult patients, the deep pelvic origin of the duplicated tract may make conventional perineal surgery technically insufficient.

We report a rare adult‐onset case of Effmann type IIA2 Y‐duplication presenting as chronic perineal wetness, highlighting key diagnostic pitfalls and describing a tailored surgical strategy using combined perineal and laparoscopic transabdominal approaches with light‐emitting catheter guidance.

## 2 Case Presentation

A man in his early 20s presented with a 3‐year history of intermittent perineal wetness. He reported no dysuria, urinary incontinence, recurrent urinary tract infections, bowel symptoms, or prior perineal trauma or surgery. He described occasional awareness that “something was coming out,” but could not determine what the fluid was or from where it originated, which caused persistent anxiety. He eventually began using pads for symptom control.

At an outside hospital, the patient noted that the wetness seemed more apparent during defecation, raising suspicion of a rectovesical or rectoperineal fistula. Comprehensive investigations—including cystoscopy, urethrocystography, contrast‐enhanced computed tomography, pelvic magnetic resonance imaging, and lower gastrointestinal endoscopy—were all normal, and no diagnosis was established. He was referred to our institution for further evaluation.

At our hospital, the first diagnostic step was to determine the nature of the discharge. Intravenous indigo carmine was administered, and blue staining of the perineal pad confirmed that the fluid was urine. Because a very small urinary outlet was suspected, detailed evaluation was planned under general anesthesia to enable simultaneous endoscopic assessment of the urinary tract and meticulous inspection of the perineum.

**FIGURE 1:**
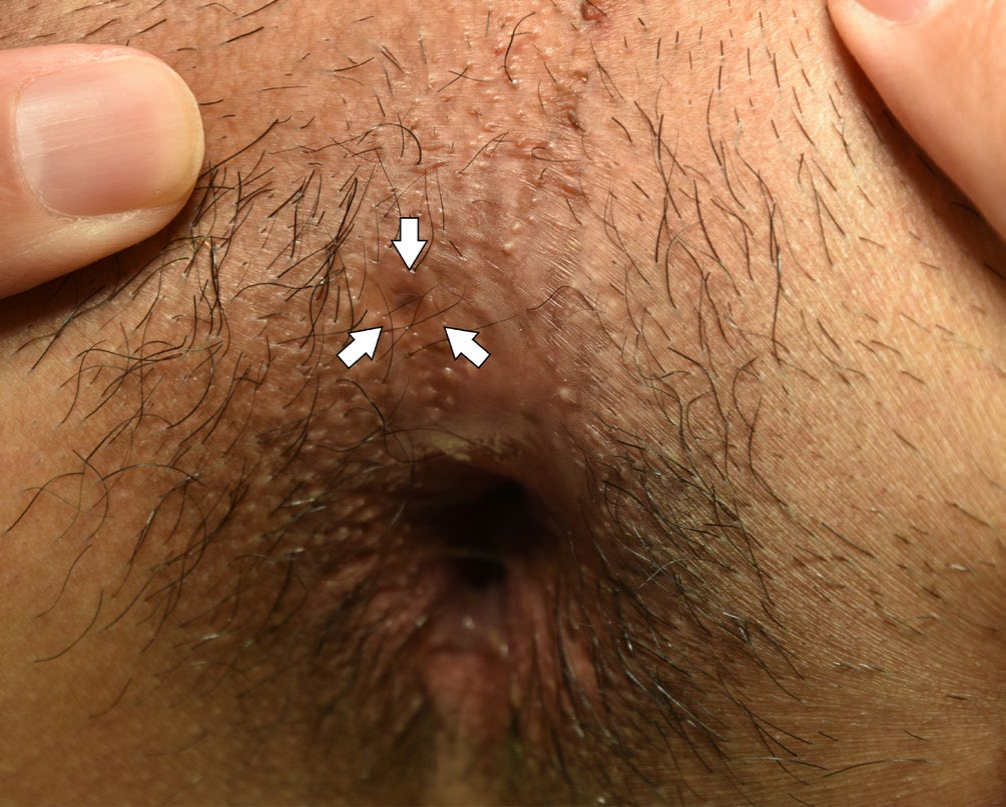
Perineal examination under general anesthesia revealing a tiny pinhole‐like opening at the 11 o'clock position near the anus (arrow).

**FIGURE 2:**
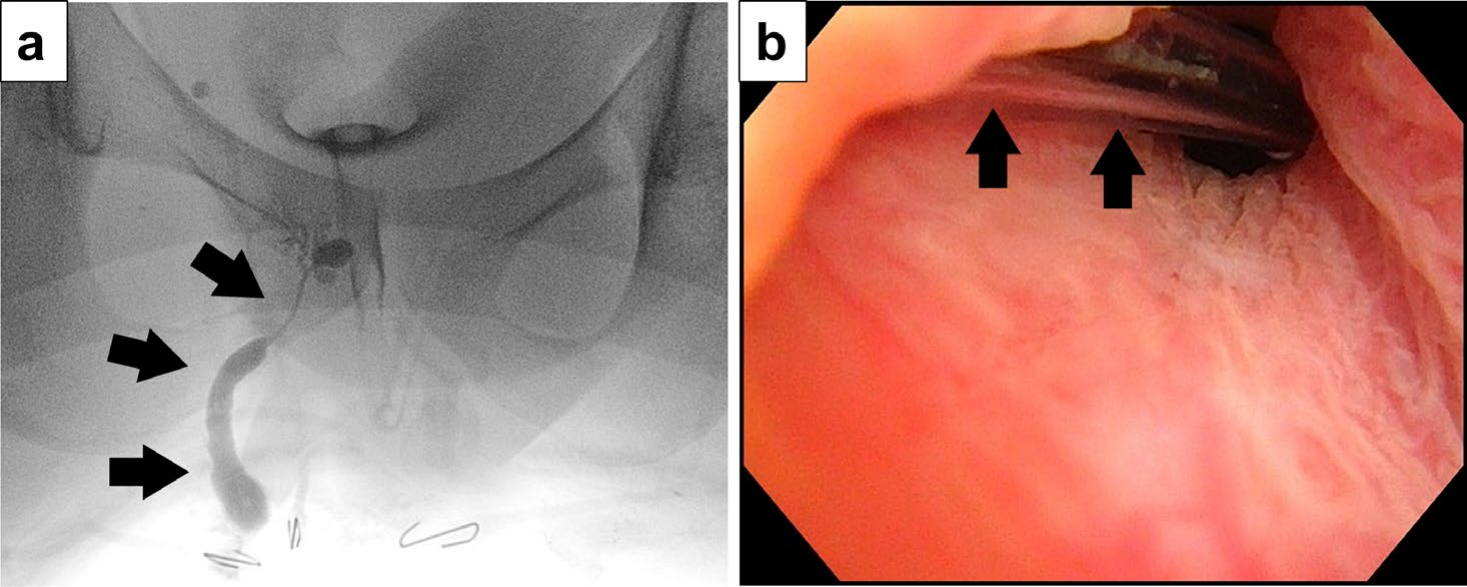
(a) Fistulogram showing contrast filling a tract extending from the perineal opening to the posterior urethra (arrow), consistent with a duplicated urethral pathway. (b) Cystoscopic image showing the catheter inserted through the perineal opening emerging from the right prostatic lobe (arrow), identifying the origin of the accessory urethra.

During cystoscopic examination of the urethra, a careful perineal inspection revealed a tiny, non‐inflamed pinhole‐like opening at the 11 o'clock position adjacent to the anal verge (Figure 1). Targeted fistulography through this opening demonstrated a narrow tract extending cranially toward the posterior urethra (Figure 2a). Subsequent cystoscopy confirmed that a catheter inserted via the perineal opening emerged from the right side of the prostatic urethra (Figure 2b), establishing the diagnosis of Effmann type IIA2 (Y‐type) urethral duplication.

**FIGURE 3:**
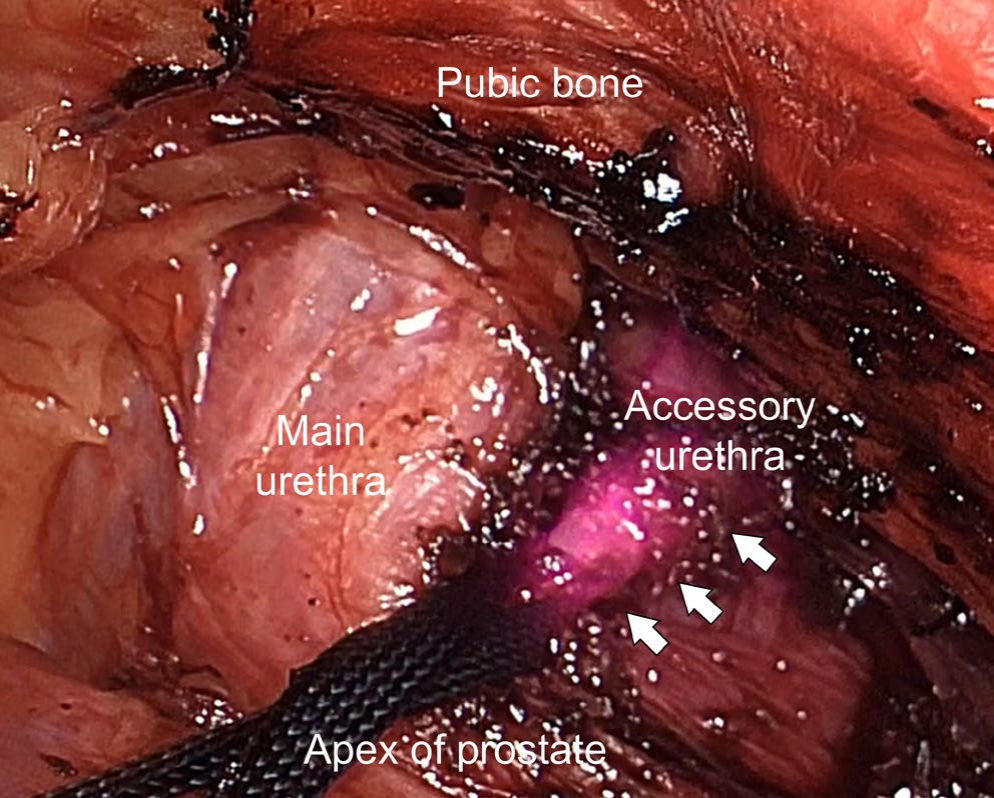
Laparoscopic image showing the fluorescent accessory urethra (arrow, encircled by a vessel loop) adjacent to the main urethra.

Definitive treatment comprised complete excision of the accessory urethra [6]. In pediatric cases diagnosed early, a perineal approach alone is frequently sufficient to reach the branching point and achieve complete resection [7]. However, in this adult patient, the accessory tract originated deep within the pelvis at the level of the prostatic urethra, and a perineal approach alone was considered insufficient to ensure secure proximal control. Therefore, a combined perineal and laparoscopic transabdominal approach was selected. A light‐emitting ureteral catheter inserted through the accessory urethra enabled continuous visualization of the tract during laparoscopic dissection, allowing secure ligation close to its origin while minimizing manipulation near the external urethral sphincter (Figure 3). The accessory urethra was completely excised without complication.

Postoperatively, perineal wetness resolved immediately. Urinary continence and erectile function were preserved, and the patient returned to normal daily activities without restriction. No recurrence or complications were observed during follow‐up.

## 3 Discussion

This case underscores the diagnostic difficulty of rare congenital urological anomalies presenting in adulthood with non‐specific symptoms. Effmann type IIA2 Y‐duplication is typically identified in childhood, and adult‐onset presentation is exceptionally rare, often resulting in prolonged diagnostic uncertainty [5].

In this patient, the initial suspicion of an enteric fistula was reasonable given the perceived association with defecation. However, repeatedly normal gastrointestinal and urological imaging highlighted the limitations of standard investigations in detecting small, non‐dilated accessory tracts. Confirming the urinary origin of the discharge using indigo carmine was a pivotal step that redirected diagnostic reasoning and enabled targeted anatomical evaluation. This emphasizes the importance of first establishing the nature of unexplained perineal discharge before repeating conventional imaging.

The anatomical configuration observed in this case is consistent with congenital posterior urethroperineal fistula, a variant within the spectrum of Effmann type IIA2 duplication [6, 8]. In such cases, a normally functioning orthotopic urethra coexists with a narrow accessory tract arising from the posterior urethra. This configuration may remain clinically silent for years, particularly if the tract remains narrow and non‐infected.

The delayed onset of symptoms in early adulthood remains incompletely understood. Histopathology revealed no significant inflammatory changes, suggesting that chronic infection was not the primary trigger. Post‐pubertal anatomical and functional changes of the prostatic urethra, along with increased urethral pressure during voiding or ejaculation, may facilitate intermittent flow into a previously silent tract, eventually resulting in persistent urinary leakage [9, 10].

Surgical management aims at complete excision of the accessory urethra to prevent recurrence of urinary leakage and avoid long‐term complications [6]. In pediatric patients, a perineal approach alone is frequently sufficient [7]. However, adult cases are rare, and the deeper pelvic origin of the duplicated tract may preclude adequate proximal control via a perineal approach alone. In this case, combining a perineal and laparoscopic transabdominal approach allowed safe access to the branching point.

Using a light‐emitting ureteral catheter provided continuous visual guidance, enabling precise dissection while minimizing unnecessary manipulation near continence‐related structures. This strategy was instrumental in achieving complete excision while preserving urinary continence and erectile function, outcomes of particular importance in adult patients.

Although rare, urethral duplication should be considered in adults with persistent perineal wetness when standard investigations are unrevealing. Careful clinical examination, confirmation of the urinary source of discharge, and targeted imaging are essential for accurate diagnosis. Surgical management should be individualized, with particular attention to anatomical depth and functional preservation.

## Ethics Statement

The authors have nothing to report.

## Consent

Written informed consent was obtained from the patient to publish this case report and accompanying images.

## Conflicts of Interest

The authors declare no conflicts of interest.

## Data Availability

Data sharing is not applicable to this article as no datasets were generated or analyzed during the current study.
